# SnS Nanoflakes/Graphene Hybrid: Towards Broadband Spectral Response and Fast Photoresponse

**DOI:** 10.3390/nano12162777

**Published:** 2022-08-13

**Authors:** Xiangyang Li, Shuangchen Ruan, Haiou Zhu

**Affiliations:** 1 College of Applied Technology, Shenzhen University, Shenzhen 518060, China; 2 College of New Energy and New Materials, Shenzhen Technology University, Shenzhen 518118, China

**Keywords:** photodetector, graphene, SnS nanoflakes, heterostructures, broadband, sensitivity

## Abstract

High responsivity has been recently achieved in a graphene-based hybrid photogating mechanism photodetector using two-dimensional (2D) semiconductor nanosheets or quantum dots (QDs) sensitizers. However, there is a major challenge of obtaining photodetectors of fast photoresponse time and broad spectral photoresponse at room temperature due to the high trap density generated at the interface of nanostructure/graphene or the large band gap of QDs. The van der Waals interfacial coupling in small bandgap 2D/graphene heterostructures has enabled broadband photodetection. However, most of the photocarriers in the hybrid structure originate from the photoconductive effect, and it is still a challenge to achieve fast photodetection. Here, we directly grow SnS nanoflakes on graphene by the physical vapor deposition (PVD) method, which can avoid contamination between SnS absorbing layer and graphene and also ensures the high quality and low trap density of SnS. The results demonstrate the extended broad-spectrum photoresponse of the photodetector over a wide spectral range from 375 nm to 1550 nm. The broadband photodetecting mechanisms based on a photogating effect induced by the transferring of photo-induced carrier and photo-hot carrier are discussed in detail. More interestingly, the device also exhibits a large photoresponsivity of 41.3 AW^−1^ and a fast response time of around 19 ms at 1550 nm. This study reveals strategies for broadband response and sensitive photodetectors with SnS nanoflakes/graphene.

## 1. Introduction

Photodetectors have attracted widespread attention over the past decade due to their potential applications in remote sensing, medical imaging, and optical communications, where broadband photodetectors with high responsivity and high sensitivity are highly required [1,2,3,4]. Photodetectors based on silicon transistor technology have attracted the interest of researchers more and more; however, the narrow absorption spectral range of silicon materials limits their application in broadband photodetectors, especially with a band larger than 1.1 μm [5]. Recently, novel 2D materials have exhibited outstanding optoelectrical properties. For example, broadband photoresponse is realized in graphene-based photodetectors due to their broadband absorption [6], and ultrafast speed operation is realized for their ultrafast carrier mobility [7]. 2D MoTe_2_ was successfully utilized in the photodetector, which shows a broadband photoresponse with a stable photocurrent distribution [8]. IV-VI compounds, such as GeSe or SnSe, also showed high efficiency from the ultraviolet (UV) to near-infrared (NIR) band range [9,10]. Hence, a new era of broadband photodetection technology based on 2D materials is emerging.

Stannous sulfide (SnS), which has a layered structure similar to graphene, is a representative p-type semiconductor of transition metal monochalcogenides [11]. The bandgap of SnS is about 1.0–1.3 eV in the mid-IR range, and the experimental absorption coefficient of SnS is about 10^4^ cm^−1^, making it an ideal material for photodetectors [12,13,14]. A SnS crystal-based photodetector was fabricated by Jethwa et al. via direct vapor transport technique, and the photodetector showed a responsivity of 0.02 mA/W at 670 nm [15]. A SnS nanosheets-based near-infrared photodetectors have been fabricated by Zhang et al. with a high responsivity of 365 A/W at 808 nm [16]. Besides SnS crystal, monolayer SnS is used in photodetector via liquid-metal exfoliation; the fabricated photodetector shows broadband spectral response from 280 nm to 850 nm, and the responsivity is 927 A/W at 660 nm [17]. The construction of heterostructures is also proved to be effective in the improvement of the responsivity of SnS-based photodetectors [18,19]. Zhao et al. designed a self-powered visible photodetector based on the CdS/SnS van der Waals heterostructure, in which the hydrothermally grown CdS nanorod arrays were covered by SnS nanoflakes. They achieved a broadband photoresponse, and the responsivity is 10.4 mA/W at 650 nm [20]. Li et al. demonstrated a one-step chemical vapor deposition (CVD) method to prepare SnS_2_/SnS vertical heterostructures, and the obtained responsivity is 27.7 A/W at 660 nm [21]. The above results strongly suggest that 2D SnS are a promising candidate for optoelectronic applications. Nevertheless, in practice, the low responsivity and narrow photoresponse range limit the practical application of the reported SnS-based photodetectors. Recently, the hot-carrier-assisted photothermal effect has been demonstrated to improve the sensitivity of 2D photodetectors for infrared light [22,23]. SnS not only exhibits an excellent photoresponse but also yields a maximum thermoelectric figure of merit ~1.36 at room temperature [24]. Therefore, in principle, an ultrabroad spectral response can be achieved by the photo carriers generated through the synergistic effect of photoconductive and photothermal effects in SnS. However, the related results are still rarely reported.

Although graphene-based photodetectors exhibit broadband photoresponse, the fast carrier recombination and weak absorption rate (2.3%) of monolayer graphene led to poor device performance, and the responsivity was limited to a few mA/W [25,26]. The graphene hybrid structures were primarily studied to improve the responsivity of graphene. In these devices, optical microcavities, waveguides, plasmonic structures, and contact engineering were integrated with graphene to enhance the responsivity through light–matter interactions in these structures [27,28,29,30,31]. Khan et al. prepared asymmetrically contacted Gr/WS_2_/Ni photodetectors, showing a photoresponsivity of 4 × 10^4^ AW^−1^ at 532 nm [30]. Li et al. fabricated a MoS_2_ photodetector with sub-30 nm channel length using graphene as source and drain, exhibiting a high optical loudness of 2.2 × 10^5^ A/W at 432 nm [31]. Furthermore, since hBN can act as a tunnel barrier and an atomically smooth substrate for graphene, the photoelectric response based on graphene/hBN/MoS_2_ van der Waals heterostructure has been demonstrated [32]. On the other hand, the introduction of additional light absorption materials at the surface of graphene appears to be a more promising approach, such as QDs, nanowires, and organic complexes [33,34,35,36,37,38]. In these hybrid structures, light-excited carriers in light-absorbing nanostructures are transferred to the graphene layer, while the oppositely charged carriers are trapped in the nanostructures. Due to the high mobility of graphene, the photocarriers transferred to graphene circulate multiple times during the lifetime of the oppositely charged carrier, resulting in a high photoconductivity gain. In the meanwhile, the oppositely charged carriers also provide a photo-gating effect on the channel of the nanostructures/graphene phototransistors. The photoresponsivity of such photodetectors can reach 10^9^ A/W [33,34,35,36]. Mukherjee et al. demonstrated a WS_2_ QDs/graphene hybrid phototransistor with an ultraviolet-visible (~365 to 633 nm) broadband photoresponse [39]. Although graphene/light absorption materials hybrid structure photodetectors have certain advantages, the stability and toxicity of QDs or organic complexes will limit their large-scale practical applications [33,40]. Additionally, it is necessary to introduce catalysts or complex ligand exchange before the preparation of nanostructures/graphene devices [41]; defects are introduced at the interface of the hybrid structure, leading to a slow response speed (second scales) [33,35,36].

Here, we propose an SnS nanoflakes/graphene heterostructure consisting of SnS nanoflakes absorber layers by PVD. The PVD method can guarantee high crystal quality and low defect density of the absorber layer and obtain high-efficiency generation capability of the photo-induced carrier. Furthermore, the introduction of defects between the graphene and SnS absorber layers can thus be avoided, which ensures ultrafast transfer of charge carriers from the absorber layer to graphene. This not only improves the response speed but also maintains the high responsivity of the photodetector due to the photo-gating effect. Our device successfully extends the detection spectrum of SnS-based photodetectors to the communication band, and it is demonstrated that hot carrier transfer in SnS nanoflakes is responsible for the photoresponse of 1550 nm. Specifically, the responsivity and detectivity were 41.28 AW^−1^ and 8.94 × 10^9^ Jones at 1550 nm, respectively, under a drain-to-source bias of 1 V. Most importantly, it has a fast light response, with the light on and off time constant as short as 19 ms.

## 2. Materials and Methods

### 2.1. Synthesis and Characterization of the SnS Nanoflakes/Graphene Heterostructure Material

Single-layer graphene films were grown on 25 μm thick copper foil (Alfa Aesar, 046986, Shanghai, China) by CVD. The SnS nanoflakes were produced directly by PVD method in tube furnace. SnS powders (5 mg, purity: 99.9%, Alfa Aesar, Shanghai, China) were placed in the central part of the heating zone of the quartz tube (approximately 750 °C) as the only evaporation source material and the target substrate was placed about 10 cm downstream away from the central part. First, argon was used to purge the quartz tube. Then the argon and hydrogen (4:1) were used as the gas carrier to transport the SnS vapor onto the top of graphene films. After a growth time of 15 min, we waited for the furnace to cool to room temperature naturally.

The surface morphology of the SnS nanoflakes/graphene heterostructure was studied by SEM (FlexSEM1000, Hitachi, Tokyo, Japan). The microstructure of SnS nanoflakes was characterized by TEM and HR-TEM (FEI Tecnai F30, Philips-FEI, Amsterdam, The Netherlands). The thickness of SnS nanoflakes was obtained using an AFM (Bruker Dimension Icon, Bruker Nano Inc., Billerica, MA, USA). The Raman and Raman mapping of SnS nanoflakes/graphene heterostructure was conducted on a Raman spectrometer (Renishaw InVia, Renishaw plc, Wotton under Edge, UK) with an excitation laser of 532 nm. The ultraviolet–visible-near infrared absorbance spectroscopy of the samples have been achieved by a Cary 5000 system (Agilent, Santa Clara, CA, USA).

### 2.2. Fabrication of the SnS Nanoflakes/Graphene Devices

The heavy p-doped silicon with 300 nm thick SiO_2_ on the surface was selected as the substrate. The electrodes (Cr:Au = 10 nm:90 nm) were deposited on the substrate using a thermal evaporation technology to define the channel with a length of 0.01 mm and a width of 2 mm. The Poly (methyl methacrylate) (PMMA) solution in anisole was spin-coated on the surface of graphene, which was grown on the copper foil. The speed was 2000 rpm with a time of 60 s. The copper substrate was etched in an ammonium persulfate solution until the PMMA-capped graphene film completely fell off from the copper substrate. Next, the film was picked up using a Si/SiO_2_ substrate with prefabricated electrodes. Then, the PMMA-capped graphene device was dried at 110° for 20 min. After that, acetone solution was used to remove the PMMA. In order to reduce the damage and contamination of the SnS nanoflakes during the subsequent transfer process, finally, the graphene device was placed downstream of the heating zone for the epitaxial growth of SnS nanosheets on intrinsic graphene. For the absorbance measurement, similarly, the graphene film was transferred on a quartz substrate for the growth of SnS nanoflakes.

### 2.3. Preparation of SnS Nanosheets (NSs) and SnS NSs/Graphene Device

Briefly, bulk SnS (50 mg) was ground in a mortar for 30 min, then added to 50 mL of N-Methylpyrrolidone solution and sonicated with a probe for 2 h. The obtained solution was subsequently sonicated in a water bath for 24 h at a frequency of 50 kHz and a power of 30 W. The supernatant was obtained by centrifugation at 4000 rpm, and then the supernatant was centrifuged at 12,000 rpm to collect the sediment, followed by washing three times with acetone and alcohol. Finally, the obtained sediment was dispersed in deionized water/alcohol mixture to form SnS NSs. The SnS NSs solution was spin-coated onto the graphene layer at 1000 rpm for 10 s, followed by drying at 80 °C for 1 h to form the SnS NSs/graphene device.

### 2.4. Optoelectronic Measurements

In this article, photoelectric measurements were performed on a probe station (CRX-6.5K, Lake Shore, Westerville, OH, USA) equipped with a semiconductor performance analyzer (B1500, Agilent, USA). A 250 W Xenon lamp coupled with the 300,150 monochromators was used as the light source to measure the optical response of the device. In order to evaluate the photoresponse characteristics of the photodetector, several independent external laser sources with different wavelengths of 375 nm, 532 nm, 980 nm, and 1550 nm were irradiated to the surface of the device by fiber coupling. The spot diameter was 4 mm. All experiments in this study were done in the air.

## 3. Results and Discussion

**Figure 1:**
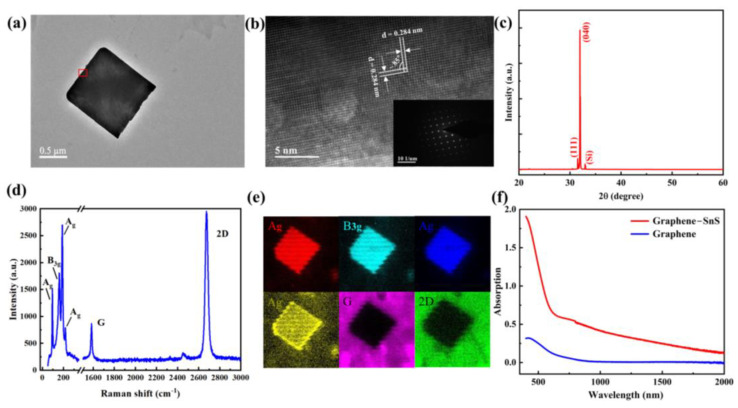
(**a**) The TEM image of a single SnS nanoflake; (**b**) The HR-TEM image of SnS nanoflakes marked by the red square in panel a; Inset: SAED pattern. (**c**) The X−ray diffraction pattern of SnS nanoflakes on SiO_2_/Si substrate. (**d**) The Raman spectra of graphene/SnS nanoflakes heterostructure. (**e**) The Raman mapping of a randomly selected graphene/SnS nanoflakes heterostructure. (**f**) The UV−Vis to NIR absorbance spectra of graphene and SnS nanoflakes/graphene heterostructure.

**Figure 2:**
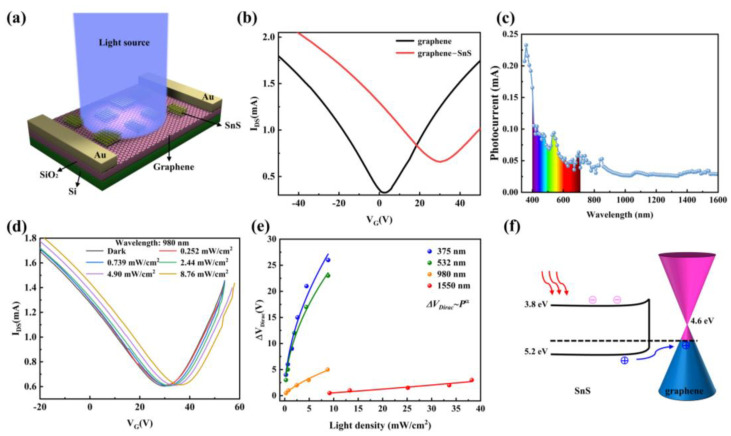
(**a**) Device characterizations of SnS nanoflakes/graphene heterostructure phototransistor. (**b**) The transfer characterization of the transistors based on pure graphene and SnS nanoflakes/graphene heterostructure in the dark, V_DS_ = 0.1 V. (**c**) Spectral response curve of the phototransistor based on SnS nanoflakes/graphene heterostructure, V_G_ = 0 V, V_DS_ = 1 V. (**d**) The transfer characteristics curve of the hybrid phototransistor at 980 nm light with different power density, V_DS_ = 0.1 V. (**e**) The summary plot of the shift of the Dirac point as a function of light power density with different wavelengths. The lines were obtained by fitting a power function. (**f**) Schematic of the photoresponse mechanism of SnS nanoflakes/graphene heterostructure.

SnS nanoflakes were epitaxially grown on the graphene film by the PVD method, as illustrated in Figure S1 (Supplementary Materials). The transmission electron microscopy (TEM) images are shown in Figure 1a,b. The lattice spacing of about 0.284 nm could be indexed to (040) planes of orthorhombic structure SnS. An angle of approximately 85° between [011] and [01$\bar{1}$] was also observed, which is consistent with the structure [11,42]. The X-ray diffraction of SnS nanoflakes is shown in Figure 1c, with two strong diffraction peaks at 30.5° and 31.9°, which correspond to the (111) and (040) planes of SnS, respectively [11]. The scanning electron microscope (SEM) picture of SnS nanoflakes/graphene heterostructure are shown in Figure S2a (Supplementary Materials), which indicates that the submicrometer SnS nanoflakes are grown uniformly on monolayer graphene. Figure S2b shows the lateral size distribution of SnS nanoflakes. The atomic force microscopy (AFM) image of as-prepared SnS nanoflakes on the graphene is shown in Figure S3 (Supplementary Materials), which reveals that the SnS nanoflakes have a thickness varied from 30 nm to 400 nm, which is suitable for the observation of optoelectronics and thermal electrons in this type of materials [43]. Raman spectra are measured to further confirm the structure of the graphene/SnS nanoflakes channel, as shown in Figure 2d. Figure S4 shows the thickness of the graphene. In addition, four characteristic Raman peaks at 94, 157, 219, and 187 cm^−1^ could be attributed to the A_g_ and B_3g_ modes of SnS nanoflakes, respectively [17]. The Raman mapping of the SnS nanoflakes/graphene heterostructure is shown in Figure 1e. The results clearly indicate the high uniformity of the SnS nanoflakes and graphene. Due to the wide and high spectral absorption of the SnS nanoflakes, as shown in the UV-Vis-NIR absorption spectrum of Figure S5 (Supplementary Materials), the absorbance of the SnS nanoflakes/graphene heterostructure was universally enhanced over a wide range of wavelengths, as shown in Figure 1f. The bandgap of SnS measured from Figure S4 (Supplementary Materials) reveals that the bandgap is about 0.98 eV, which corresponds to an absorption edge of 1265 nm.

A 3D schematic of the photodetector based on graphene/SnS nanoflakes heterostructure is illustrated in Figure 2a. Two Au electrodes are thermally evaporated onto the substrate to define source/drain, which are in contact with graphene. Besides, we prepared pure graphene devices with the same configuration for comparison purposes. The optical image of graphene/SnS nanoflakes heterostructure device is shown in Figure S6 (Supplementary Materials). The Dirac point in the graphene transistor is located at +3 V, which may be assigned to the modification by the substrate or water and oxygen in the atmosphere trapped beneath the graphene film [44]. The field-effect mobilities of the graphene film are ~173 cm^2^s^−1^V^−1^. The Dirac point of the SnS nanoflakes/graphene heterostructure transistor device shifts to a more positive position than the pure graphene device. This implies a p-type doping effect caused by SnS nanoflakes to graphene, together with a slight increase in the minimum conductivity [45]. The energy level positions of the conduction and valence bands of SnS nanoflakes are 3.8 and 5.2 eV, respectively [21]. Furthermore, SnS is a p-type semiconductor with Fermi energy close to its valence band [21]. The Fermi level of intrinsic graphene is about 4.6 eV [33], as shown in Figure S7. Therefore, after contact, the holes in SnS nanoflakes will transfer to the graphene film and the graphene become more p-type [22,43]. The transfer of holes from SnS nanoflakes to graphene leads to band bending in SnS to balance the Fermi level. In addition, the reduction of the Fermi level of graphene results in a larger contact resistance for electron injection [33]. The mobility of electrons decreases to ~87 cm^2^s^−1^V^−1^, but the mobility of the holes is almost unchanged. In order to demonstrate the typical broadband photoresponse characteristics of the SnS nanoflakes/graphene hybrid device, we first explored the spectral response curve from 350 nm to near-infrared under V_DS_ = 1 V and V_G_ = 0 V, as shown in Figure 2c. The peak photocurrent was observed at approximately 532 nm, which may be due to the relatively high power of the light source at that specific wavelength, as shown in Figure S8. More importantly, the curve trend was well consistent with the absorbance spectrum of the SnS nanoflakes/graphene film (Figure 1f). Thus, it can be inferred that the broadband response-ability was mainly attributed to the light-harvesting of the hybrid film. The transfer curves of the SnS nanoflakes/graphene phototransistor under 980 nm irradiation with different light power densities are shown in Figure 2d. With the increase of light power density, the Dirac point of the hybrid device shifted positively, which indicates that the p-doping effect is enhanced. Under the irradiation of 375, 532, and 1550 nm lasers, the transfer curves of the hybrid device show the same characteristics, as shown in Figure S9 (Supplementary Materials). In comparison, the transfer characteristic curves of the pure graphene device show no obvious light response under the largest radiant fluxes of four lasers. Therefore, the photoresponse was mainly caused by SnS nanoflakes. The shifts of the Dirac points (ΔV_Dirac_) as a function of light power density (*P*) are plotted in Figure 2e for all four lasers. The curves can be fitted by the following relationship:

(1) $$\Delta V_{\mathrm{Dirac}}=AP^{\alpha },$$

where *A* and *α* are constants and *P* is light power density. It is notable that this equation has been widely used for photogating phototransistors [35,45]. The *α* values for all four lasers were obtained by fitting the experimental data are listed in Table S1 (see in Supplementary Materials). In order to explain this photosensitivity mechanism, the schematic diagram of the energy band diagram of the interface of SnS nanoflakes/graphene is shown in Figure 2f. SnS nanoflakes are responsible for light collection and for the generation of photo-induced carriers, photo-generated electrons and holes should be transferred from SnS to graphene. However, due to the energy barrier and quantum confinement at the graphene/SnS nanoflakes interface, the transfer of electrons is either forbidden or hindered, and holes are transferred to graphene [37,45]. Furthermore, the photogenerated electrons are confined in nanoflakes for a long time, so the negatively charged nanoflakes induce positive carriers in graphene through capacitive coupling, which further explains the p-doping in graphene films. As a result, the photogenerated holes transferred to the graphene will quickly move to the electrode. As long as one hole moves to the electrode, a new hole will be transferred to the graphene [41]. Due to the combination of the high carrier mobility in the graphene channel, the drain-source bias voltage V_DS_, narrow channel length, and the long confinement time of electrons in SnS nanoflakes, the holes will circulate multiple times to generate high photocurrent gain [35].

**Figure 3:**
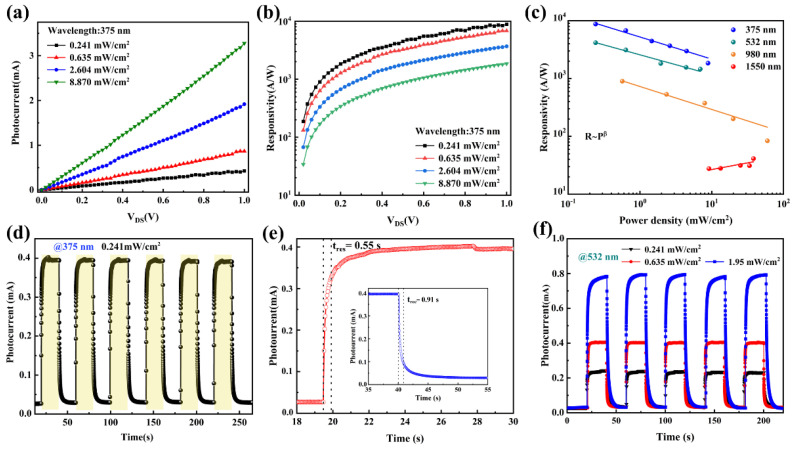
(**a**) Photocurrent and (**b**) responsivities vs. source-drain voltage curves at different light power densities; Wavelength: 375 nm; V_G_ = 0 V. (**c**) Relationships of responsivity and light power density at different light wavelength. Balls are the experimental data of responsivity, which are abstracted from the output characteristics; V_G_ = 0 V, V_DS_ = 0.1 V. (**d**) Time-dependent photocurrent of the photodetector at incident light wavelengths of 375 nm; excitation power: 0.241 mW/cm^2^, V_G_ = 0 V, V_DS_ = 0.1 V. (**e**) Single-cycle time-dependent photocurrent for rising/falling time analysis in figure d. (**f**) Time-dependent photocurrent of the photodetector at incident light wavelengths of 532 nm.

By irradiating the phototransistor with different specific wavelengths, we can accurately demonstrate the broadband detection capability and high responsivity of the device. Firstly, we studied the photo response under the irradiation of an ultraviolet light source (wavelength: 375 nm). The output characteristics curves of the devices under different light irradiation of the 375 nm are shown in Figure 3a. The photocurrent *I*_ph_ is defined as *I*_ph_ = *I*_laser_ − *I*_dark_, where *I*_dark_ and *I*_laser_ are the channel current in dark and light irradiation conditions, respectively. Here, the responsivity (*R*) represents the response of the photodetector to incident light, which can be calculated on the following relationship:

*R* = *I_ph_*/*P*, (2)

The photocurrent and voltage curves of the device show the linear characteristics and the photocurrents increase linearly with the increase of laser power density. Moreover, the photocurrent versus V_DS_ of the device under the illumination of visible (532 nm) and near-infrared (NIR: 980 nm, 1550 nm) are shown in Figure S10 (Supplementary Materials). The photocurrent of pure graphene was measured at maximum power at different wavelengths, and the maximum photocurrent of pure graphene is only 9 μA at 375 nm, as shown in Figure S11 (Supplementary Materials). The *R* of the device as a function of V_DS_ under varying intensities is displayed in Figure 3b. The responsivity increases gradually with the increase of V_DS_, which is commonly observed in hybrid graphene photodetectors [31,42,45,46]. At the largest V_DS_ of 1 V, due to the weak recombination effect of photogenerated carriers under weak light, the maximum responsivity reaches about 8.8 × 10^3^ AW^−1^ at the light intensities of 0.241 mW/cm^2^. The photoconductive gain (*G*) can be calculated by the following equation:

(3) $$G=\frac{\tau }{t_{L}},$$

where *τ* is the photocarrier lifetime, *t_L_* is the transit time of the carrier (see Note S1 in the Supplementary Materials for detail). Thus, the photoconductive gain of 4.79 × 10^7^ can be obtained at 375 nm.

Where Δ*I* and *P* represent the photocurrent and incident laser power in the channel, respectively. In addition, the photocurrent ΔI can be defined by the following equation [35]:

(4) $$\Delta I\;=\;\frac{W}{L}C_{i}\mu \Delta V_{Dirac}V_{DS},$$

where $C_{i}$ is the capacitance of the gate dielectric per unit area, *W* and *L* are the width and length of the channel, respectively. The *R* is defined by [46]:

(5) $$R\;=\;\frac{W}{LP}C_{i}\mu \Delta V_{Dirac}V_{DS}\;\frac{V_{DS}WC_{i}\mu A}{L}\;P^{\alpha -1}\;=\;BP^{\beta },$$

where *B* = $\frac{V_{DS}WC_{i}\mu A}{L}$ is a constant, and the β = α−1. Figure 3c shows the plot of *R* as a function of light power density under the irradiation of different light wavelengths. The *R* exhibits a strong dependence on light power density, it decreases with the increase of the power density under the irradiation of 375 nm, 532 nm, and 980 nm lasers when the energy of the laser is greater than the bandgap, and under the irradiation of communication band of 1550 nm, the *R* increases with the increase of laser density. The results of β are also listed in Table 1. The theoretical data of *α* and *β* are in good agreement with the experimental data with a smaller deviation.

**Figure 4:**
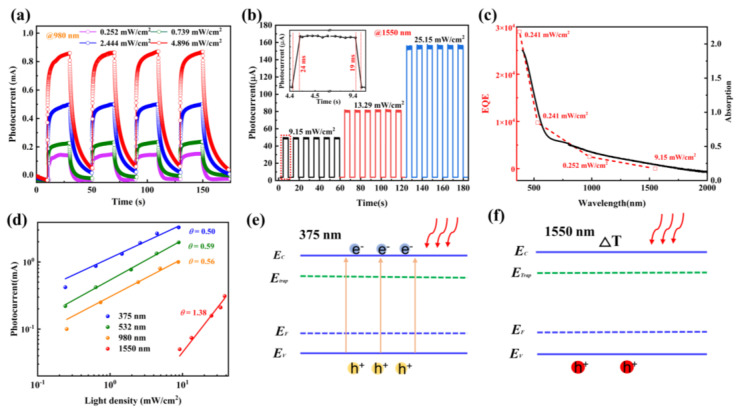
Time−dependent photocurrent of the photodetector at different incident light wavelengths of (**a**) 980 nm and (**b**) 1550 nm under various incident light power densities. (**c**) Absorbance spectrum of the SnS nanoflakes/graphene heterostructure and the spectral distribution of EQEs of the SnS nanoflakes/graphene photodetector. (**d**) Plot of photocurrents of the photodetector as a function of light power density under different laser wavelengths. Inset (**d**): Single−cycle dynamic response curve under 1550 nm; excitation power: 9.15 mW/cm^2^. Mechanism of generating photogenerated carriers based on photoconductivity effects (**e**) and photothermal effects (**f**).

The time-dependent photocurrent for multiple irradiation cycles at a constant light density of 375 nm with V_DS_ = 1 V and incident power intensity of 0.241 mW/cm^2^ are displayed in Figure 3d. The photocurrent shows a steady enhancement and good repeatability of photoswitching. In addition, it can be seen that the photocurrent presents four stages in each on/off cycle of the laser, including continuous sharp rise, slow rising, sharp falling, and slowly falling. This is the typical photoresponse characteristic of photoconductive detectors, as the photon energy (~3.3 eV) of 375 nm light is much higher than the bandgap of SnS. Thus, the energy of the laser is enough for the separation of electron-hole pairs [23,46,47]. Response time is defined as the time interval from 10% to 90% of the peak value in a single cycle [48]. Figure 3e shows a single-cycle time-dependent photocurrent curve at 375 nm, showing a rising time of ~0.55 s and a falling time of ~0.91 s, respectively. Figure 3f shows the photoswitching curves at the irradiation wavelengths of 532 nm (V_DS_  =  1 V). Similar to photoresponse under 375 nm laser, the device exhibits obvious four-stage dynamic behavior under irradiation, indicating the photoconductive response. In addition, the photosensitivity behavior can be modulated by increasing the laser power density. The performance of the graphene/SnS nanoflakes heterostructure photodetector at the NIR region was further investigated. Figure 4a presents the photoswitching curves under the irradiation of laser of 980 nm at V_DS_  =  1 V. Due to the wide-spectrum absorption of SnS nanoflakes, the device also exhibits obvious four-stage dynamic behavior under the irradiation of 980 nm. The dynamic optical response curves of the device under laser irradiation of 1550 nm were also studied. It should be noted that the energy of 1550 nm is lower than the bandgap of SnS nanoflakes. Similar to irradiation with other wavelengths, the photocurrent can be effectively turned on and off and modulated by different laser powers. However, the photodetector only exhibits two-level response characteristics, with a steep rise/fall in each cycle, and the rising/falling speeds are 24 ms/19 ms (Figure 4b inset) respectively, showing a much higher time resolution. Several recent reports on graphene-based hybrid photodetectors are compared (see in Table 1), and the response rising time in our device is comparable to the 10–20 ms reported in the CVD graphene/GaSe-nanosheets hybrid phototransistor [48]. The corresponding decay time shows significant improvement when compared with the 100 ms reported in the exfoliated graphene/PbS-QDs phototransistor and the few seconds reported in the CVD graphene-hybrid [32,33,34,35,45,46]. In order to further study the broadband response capability of the photodetector, the external quantum efficiencies (*EQEs*) of the device under the four lasers were calculated by the equation *EQEs* = *Rhν*/*e*, where *h* is the Planck constant, *ν* is the light frequency, and *e* is the elementary charge. Considering the dependence of *EQEs* on photon wavelength, the *EQEs* were calculated using the most comparable light power density. The calculation is used to simulate the absorbance of the graphene/SnS nanoflakes heterostructure, as shown in Figure 4c, which is well matched.

The fitting plots of the photocurrent as a function of laser power density at different wavelengths (including 375 nm, 532 nm, 980 nm, and 1550 nm) are shown in Figure 4d. The relationship between the incident laser power density and the photocurrent can be fitted to the law *I_ph_ = AP^θ^*, where *A* is a constant, *θ* is the index of the light response at a specific wavelength [49]. In this study, the value of *θ* varied from 0.50 to 0.61, and the ideal factor increases to 1.38 in the communication band of 1550 nm. Due to defects and charge impurities in the materials or the existence of adsorbed molecules at the interface, for photoconductor devices, the photocurrent and power-dependent ideality factor *θ* is generally less than 1 [50]. The dramatic increase of the ideal factor means that there is now another reaction mechanism at work [51]. Since the photon energy at 1550 nm is lower than the bandgap of SnS nanoflakes, this means that the photoconductivity effect cannot be dominant. In addition, the photocurrent of the device under laser irradiation must be realized under an external voltage, so we attribute it to the photothermal effect [52]. In order to further clarify the difference in the photoresponse mechanism, we built an energy band model. The photoresponse mechanism of photoconductive effect and photothermal effect are shown schematically in Figure 4e,f. When the device receives an optical signal with photon energy higher than the bandgap, such as a photon of 375 nm, the photon can be absorbed by the SnS nanoflakes and generate light-excited electron-hole pairs. Due to the efficient interfacial coupling between SnS nanoflakes and graphene, the high carrier mobility of graphene, and the smaller channel length, light-excited holes will be quickly transferred to graphene and then cycled many times during the limited lifetime of electrons, thereby generating photocurrent. However, there will inevitably be some local defects in the SnS nanoflakes, such as tin vacancy [53]. Figure S12 (Supplementary Materials) shows the EDS results of the SnS nanoflakes, indicating a slight tin defect. These local defects, which are located below the conduction band (E_c_) will form defect energy levels (E_traps_), and they can capture photo-excited electrons in the valence band [54]. This electron capture/release is a slower process. Therefore, there is a slow rising/falling stage in the transient photoresponse of our device. Figure S13a (Supplementary Materials) shows the image of the SnS nanoflakes device. Figure S13b (Supplementary Materials) presents the source-drain curves of the SnS nanoflakes device with different temperatures, showing sub-linear and asymmetric. The resistance of SnS nanoflakes gradually decreases with the increase of temperature. The photoresponse of the SnS nanoflakes was also shown in Figure S14. In combination with the research results of others [35,55], there is a strong bolometric effect in the SnS nanoflakes, similar to SnSe [23]. The transport characteristics of the SnS nanoflakes device at V_DS_ = 0.1 V are shown in Figure S15 (Supplementary Materials). The current in the source-drain decreases with the increase of gated voltage, showing an apparent p-type behavior, and the holes dominate the charge transport processes. Based on this strong bolometric effect, the hole concentration in SnS nanoflakes is caused by ΔT under the irradiation of the 1550 nm lasers, as shown in Figure 4e. The holes transferred to the graphene film are different from the photo-excited e–h pairs but are the photothermal-induced hole-type carriers in the valence band of SnS nanoflakes. Thus, the photobolometric effect does not involve the carrier capture and releasing process. As shown in Figure 4b, the device exhibits a faster optical response time after the laser is switched on/off, and there is no moderate rising/falling process in each cycle. The narrow channel length (10 μm) of our device is also beneficial to improve the response time, which can be attributed to the reduction of recombination and scattering during the transport of photogenerated carriers [56]. Figure S16 shows the rise/decay response versus light power density of SnS nanoflakes/graphene devices at different wavelengths. The results show that the device has the fastest response speed at 1550 nm due to the transfer of photothermal carriers, and the rise/decay response of the device is almost constant. However, due to the photoconductive effect, the rise/decay response of the device shows an increasing trend with the increasing laser power under the laser of 375 nm, 532 nm, and 980 nm.

Due to the enhancement of the photothermal effect under the strong light of 1550 nm, the R increases with increasing laser power density (Figure 3c). However, the optical response of photothermal effect is much weaker than the photoconductor effect. For example, R is 41.3 AW^−1^ for 1550 nm, which is much lower than 8.8 × 10^3^ AW^−1^ for 375 nm and 2.0 × 10^3^ AW^−1^ for 980 nm. In addition, there is almost no apparent photogating effect under the light of 1550 nm because of the lack of electron restriction in the SnS nanoflakes (Figure S9c). The noise spectral density of a phototransistor is composed of three kinds of noise (1/*f* noise, shot noise, thermal noise). The noise equivalent power (*NEP*) value can be calculated from *NEP* = *S_I_*/*R* and the *S_I_* is the total current noise [39] (see Note S2 in the Supplementary Materials for detail). The results show that the dark current of our device is dominated by 1/*f* noise. The value of 1/*f* noise is 4.85 × 10^−15^ A^2^/Hz at a modulation frequency of 1 Hz, as shown in Figure S17. The detectivity can be used to evaluate the device’s ability to detect weak light signals. This parameter can be calculated by the equation [48]:

(6) $$D*\;=\;\frac{R\sqrt{A}}{\sqrt{2qI_{d}}},$$

where $I_{d}$ and *A* are the dark current and effective channel area, respectively. The power-dependent detectivity under different laser wavelengths is shown in Figure S10d (Supplementary Materials). From our results, the SnS nanoflakes/graphene photodetector shows the highest responsivity of 8.8 × 10^3^ AW^−1^ with the corresponding detectivity of 1.9 × 10^12^ cm Hz^1/2^ W^−1^ (Jones) at the wavelength of 375 nm. Under 1550 nm laser, a high responsivity of R = 41.3 AW^−1^ with a detectivity of D* = 8.9 × 10^9^ Jones can be achieved, which is ∼2 orders of magnitude larger than other reported ones [23,57,58,59]. It should be noted that the distribution density of SnS nanoflakes can affect the photocurrent of photodetectors (Figure S18, Supplementary Materials). Furthermore, the reliability of the results was checked by measuring a second device, which was synthesized under the same conditions. The results show that there are no significant differences in photocurrent (Figure S19, Supplementary Materials).

**Table 1:** Comparison of the recent graphene/nanostructure and 2D SnS photodetectors.

Structure	R (A/W)	Response Time	Light	Reference
Au/SnS nanosheets	635	τ_ON_ ~350 ms τ_OFF_ ~350 ms	808 nm V_DS_ = 1 V	[16]
Graphene/PbS QDs	5 × 10^7^	τ_ON_ ~0.3 s τ_OFF_ ~1.7 s	895 nm V_DS_ = 5 V	[34]
Graphene/ZnO	1.89 × 10^6^	No reported	365 nm V_DS_ = 5 V	[35]
Graphene/GaSe	10^5^	τ_ON_ ~10.0 ms τ_OFF_ ~12.8 ms	532 nm V_DS_ = 1 V	[44]
Graphene/BP nanosheets	7.7 × 10^3^	τ_ON_ ~3.0 s τ_OFF_ ~7.9 s	360–785 nm V_DS_ = 5 V	[45]
Graphene/Carbon nitride	~10^3^	τ_ON_ ~0.74 s τ_OFF_ ~8.21 s	370 nm V_DS_ = 5 V	[46]
Graphene/Bi_2_Te_3_	35	not specified, estimated τ_ON_ < 0.1 s τ_OFF_ < 0.1 s	532–1550 nm V_DS_ = 1 V	[58]
Graphene/Ge	66.2	τ_ON_ ~5.6 ms τ_OFF_ ~3.5 ms	350–1650 nm V_DS_ = 1 V	[59]
Graphene/graphene QDs	5 × 10^7^	not specified, estimated τ_ON_ < 5.0 s τ_OFF_ < 10.0 s	UV V_DS_ = 1 V	[38]
SnS nanoplates	198	τ_ON_ ~40 ms τ_OFF_ ~40 ms	405 nm–808 nm V_DS_ = 1 V	[14]
Graphene/SnS nanoflakes	8.8 × 10^3^	τ_ON_ ~24 ms τ_OFF_ ~19 ms	375–1550 nm V_DS_ = 1 V	This work

**Figure 5:**
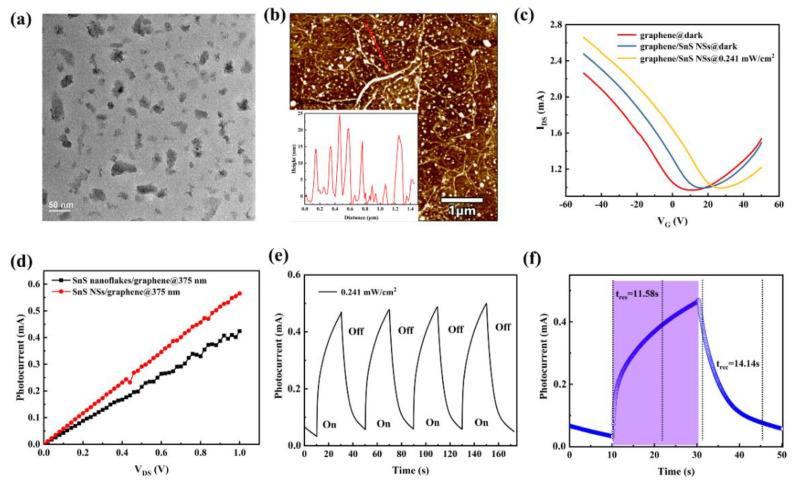
Morphology characterization of SnS NSs. (**a**) TEM image; (**b**) AFM image of the SnS NSs/graphene hybrids with a marked line; Inset: thickness profiles corresponding to a line in (**b**). (**c**) The transfer characteristics of the pure graphene device and SnS NSs/graphene hybrids device in the dark and light. (**d**) The photocurrent of the SnS NSs/graphene hybrids device and SnS nanoflakes/graphene hybrids device. (**e**) The time−dependent photocurrent for multiple irradiation cycles of the SnS NSs/graphene hybrids device. (**f**) The single time−dependent photocurrent in (**e**). (Wavelength: 375 nm; P = 0.241 mW/cm^2^).

To demonstrate the high quality and low trapped state of the SnS nanoflakes/graphene interface prepared by PVD, we fabricated an SnS NSs/graphene hybrid device by a spin coating method. The TEM image in Figure 5a reveals the distributed lateral dimensions of the SnS NSs. Furthermore, the HR-TEM image of SnS NSs in Figure S20 (Supplementary Materials) further shows a lattice spacing of 0.284 nm and a crystal plane angle of 85°, which is consistent with the results of SnS nanoflakes (Figure 1b). Figure 5b shows the AFM image of the SnS NSs/graphene hybrids, showing that the SnS NSs are uniformly coated on the surface of graphene. The corresponding thickness distribution of SnS NSs is shown in Figure 5b inset. Figure 5b shows the transfer characteristics of the transistors based on pure graphene device and SnS NSs/graphene hybrids device in the dark and light. The results show that the Dirac point of the SnS NSs/graphene hybrids device moves to positive gate voltage under dark, which is also consistent with the SnS nanoflakes/graphene device. However, electrons can be easily captured by the stacking boundaries and abundant trap states in defects of liquid-exfoliated SnS NSs [60,61,62,63]. Therefore, the offset of the Dirac point of the SnS NSs/graphene hybrids device is about 10 V, which is significantly larger than that of SnS nanoflakes/graphene (+4 V). Furthermore, due to the high trapping states in SnS NSs, the holes will circulate multiple times to generate high photocurrent gain. Thus, the photocurrent of the SnS NSs/graphene hybrids device is also larger than that of the SnS nanoflakes/graphene device, as shown in Figure 5d. Figure 5e,f shows the time-dependent photocurrent for multiple and single irradiation cycles at a constant light of 375 nm with V_DS_ = 1 V. It has been demonstrated that the high quality between graphene and nanostructure can significantly improve the response speed of the hybrid device [41,58,59]. However, the result of the SnS NSs/graphene hybrids device shows a rising time of ~11.58 s and a falling time of ~14.14 s, respectively, which is significantly an order of magnitude slower than that of SnS nanoflakes/graphene.

## 4. Conclusions

In summary, we have developed an SnS nanoflakes/graphene hybrid phototransistor for photodetection. By combining the respective unique properties of SnS and graphene, the hybrid device provides excellent gate tunability and broad spectral response from 365 nm to 1550 nm. The hybrid device also exhibits high detectivity (~1.9 × 10^12^ Jones) and moderately responsivity (~8.8 × 10^3^ AW^−1^). Most importantly is that there are fast response time constants around 19 ms and high responsivity around 41.3 AW^−1^ at 1550 nm, which are better than previous graphene-based hybrid photodetectors [56,57]. The key to achieving superior performance in this work are the synergistic effect of the photoconductive effect and the photothermal effect of the hybrid device in which SnS nanoflakes are integrated on the surface of the graphene. These findings open new pathways to design and develop broadband photodetectors based on SnS.

## Data Availability

The data presented in this study are available on request from the corresponding author.

## Supplementary Materials

The following supporting information can be downloaded at: https://www.mdpi.com/article/10.3390/nano12162777/s1, Figure S1: Schematic diagram of the experimental device of PVD method; Figure S2: (**a**) The SEM image of part of the channel area of the SnS nanoflakes/graphene device. (**b**) The size distribution of SnS nanoflakes in Figure S2a. Figure S3: (**a**) The AFM topography of SnS nanoplatelets on graphene (**b**) AFM height profile corresponding to the two dash lines in figure S3a. Figure S4: (**a**) The AFM image of the graphene film. (**b**) The thickness of the graphene. Figure S5: The UV−Vis to NIR absorption spectrum of SnS nanocrystal on quaetz substrate. Figure S6: The optical image of graphene/SnS nanoflakes heterostructure device (7 mm × 7 mm). Figure S7: The band alignment of the SnS/graphene heterostructure before contact. Figure S8: The relationship between the power distribution of the light source and the wavelength from 350 nm to 1100 nm. Figure S9: Transfer characteristics (V_DS_ = 0.1 V) of the phototransistor based on graphene−SnS nanocrystal heterostructure at different light power density with the wavelengths of (**a**) 532 nm, (**b**) 980 nm, and (**c**) 1550 nm; (**d**) Transfer characteristics (V_DS_ = 0.5 V) of the only graphene phototransistor at four different light sources with each the highest intensity. Figure S10: Photocurrent as functions of V_DS_ based on graphene-SnS nanoflakes heterostructure phototransistor at different light power density with the wavelengths of (**a**) 532 nm, (**b**) 980 nm, and (**c**) 1550 nm; (**d**) Detectivity of the device as a function of laser power densities under different laser wavelengths. Figure S11: The photocurrent of pure graphene devices under four lasers. Figure S12: EDS results of the SnS nanoflakes. Figure S13: (**a**) The SEM image of the SnS nanoflakes FET. (**b**) The source−drain curves of the SnS nanoflakes FET were measured within the temperature range of 140 K to 300 K. Figure S14: (**a**) IV curves of SnS FET under dark and various wavelengths of light with different light intensities. The photoresponse of the device under (**b**) 375 nm, (**c**) 532 nm, (**d**) 980 nm, (**e**) 1550 nm, and (**f**) The current on−off ratio of SnS FET under different wavelengths. (V_DS_ = 5 V). Figure S15: Transfer characteristic curves of the SnS nanoflake-based field-effect transistors at V_DS_ = 0.1 V. Figure S16: The power-dependent and wavelength of rise/decay response of the SnS nanoflakes/graphene devices. Figure S17. Noise spectral density of 1/*f* noise vs frequency at V_DS_ = 1 V. Figure S18: The SEM images of different amounts of SnS nanoflakes by varying the weight of (**a**) 1.0 mg, (**b**) 2.5 mg, (**c**) 5.0 mg and (**d**) 10 mg of the SnS powders, scale bar: 10 µm. (**e**) The photocurrent of the corresponding graphene-SnS nanoflakes heterostructure phototransistor. Figure S19: The photocurrent of another SnS nanoflakes/graphene devices under the laser of (**a**) 375 nm; (**b**) 532 nm; (**c**) 980 nm, and (**d**) 1550 nm. Figure S20: The HRTEM image of SnS NSs. Table S1: *α* and *β* were obtained by fitting the relationship between the *∆V_Dirac_−P* and *R−P* curves and the power function. Note S1: The calculation of photoconductive gain (G). Note S2: The calculation of *NEP.*
