# Supplementary material for: SnS Nanoflakes/Graphene Hybrid: Towards Broadband Spectral Response and Fast Photoresponse
Source: Nanomaterials (Basel). 2022 Aug 13;12(16):2777. doi: 10.3390/nano12162777 (PMC9413584; doi:10.3390/nano12162777)
Supplement: Supplementary file 1 [file nanomaterials-12-02777-s001.zip › nanomaterials-1840950-supplementary.pdf]

nanomaterials

Supplementary Materials

SnS Nanoflakes/Graphene Hybrid: Towards Broadband Spectral Response and Fast Photoresponse

Xiangyang Li [1], Shuangchen Ruan [2] and Haiou Zhu [2,*]

[1] College of Applied Technology, Shenzhen University, Shenzhen 518060, China
[2] College of New Energy and New Materials, Shenzhen Technology University, Shenzhen 518118, China
* Correspondence: zhuhaiou@sztu.edu.cn

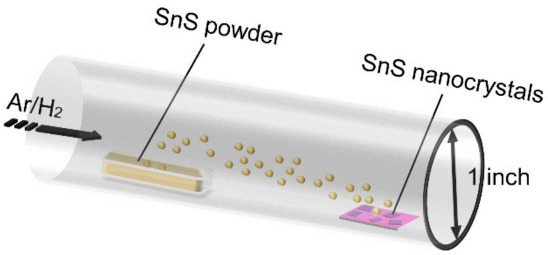

Figure S1. Schematic diagram of the experimental device of PVD method.

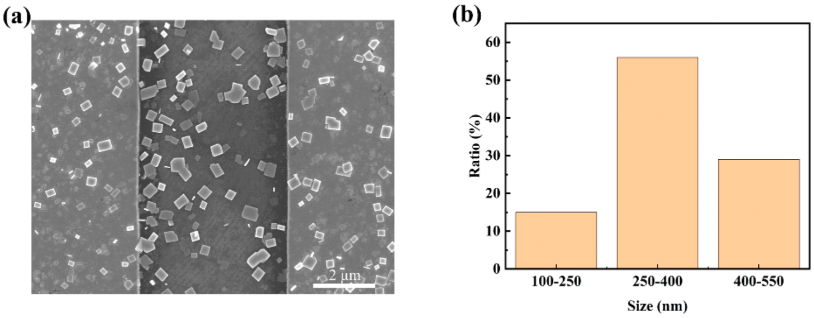

Figure S2. (**a**) The SEM image of part of the channel area of the SnS nanoflakes/graphene device. (**b**) The size distribution of SnS nanoflakes in Figure S2a.

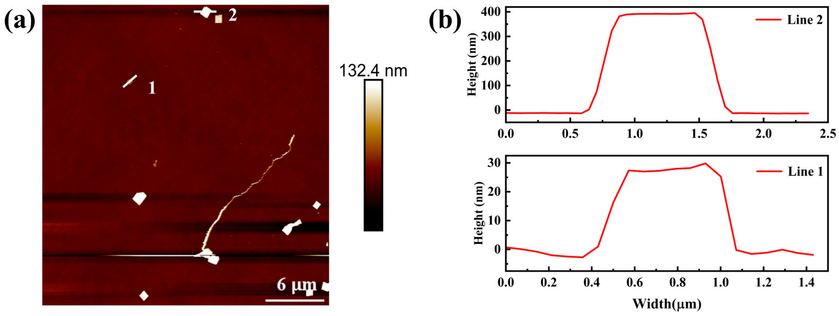

Figure S3. (**a**) The AFM topography of SnS nanoplatelets on graphene (**b**) AFM height profile corresponding to the two dash lines in figure S3a.

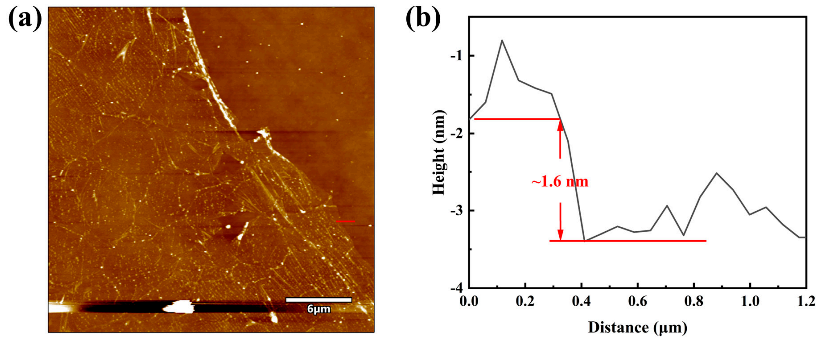

Figure S4. (**a**) The AFM image of the graphene film. (**b**) The thickness of the graphene.

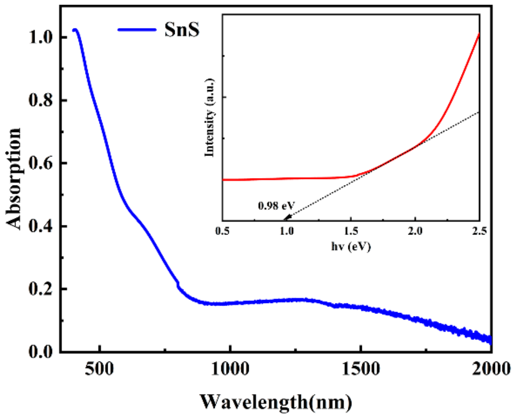

Figure S5. The UV-Vis to NIR absorption spectrum of SnS nanocrystal on quaetz substrate.

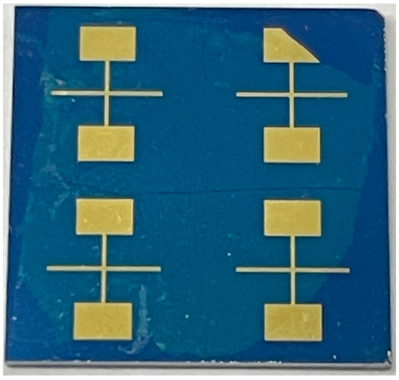

Figure S6. The optical image of graphene/SnS nanoflakes heterostructure device (7mm*7mm).

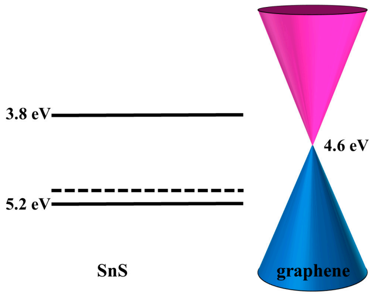

Figure S7. The band alignment of the SnS/graphene heterostructure before contact.

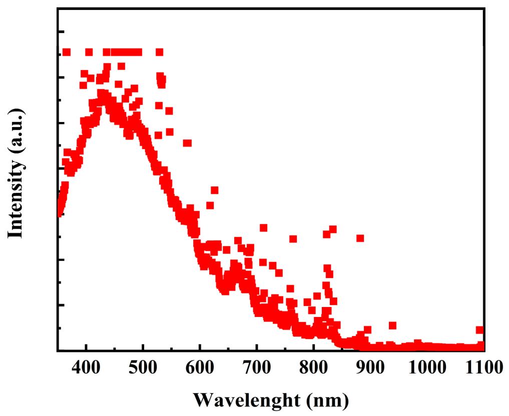

Figure S8. The relationship between the power distribution of the light source and the wavelength from 350 nm to 1100 nm.

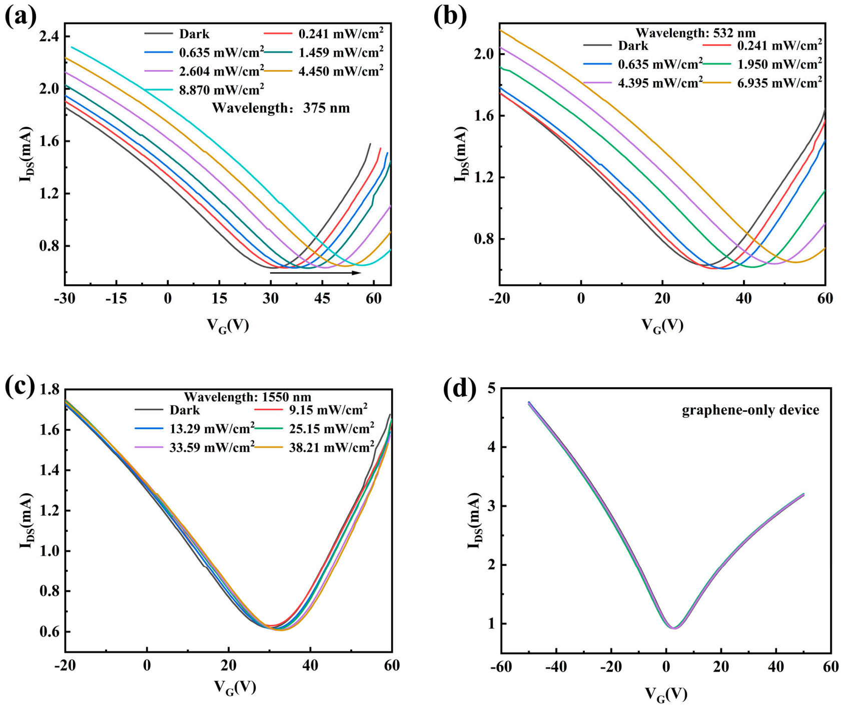

Figure S9. Transfer characteristics (V_{DS}=0.1 V) of the phototransistor based on graphene-SnS nanocrystal heterostructure at different light power density with the wavelengths of (**a**) 532 nm, (**b**) 980 nm, and (**c**) 1550 nm; (**d**) Transfer characteristics (V_{DS}=0.5 V) of the only graphene phototransistor at four different light sources with each the highest intensity.

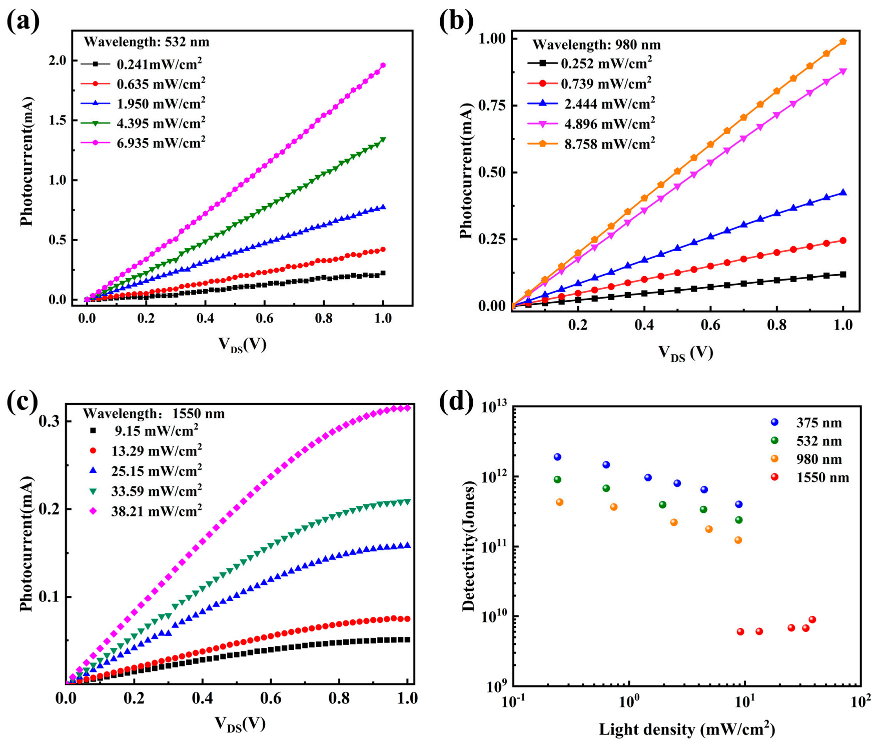

Figure S10. Photocurrent as functions of V_{DS} based on graphene-SnS nanoflakes heterostructure phototransistor at different light power density with the wavelengths of (**a**) 532 nm, (**b**) 980 nm, and (**c**) 1550 nm; (**d**) Detectivity of the device as a function of laser power densities under different laser wavelengths.

Table S1. α and β were obtained by fitting the relationship between the ΔV_{Dirac}-P and R-P curves and the power function.

Wavelength	375 nm	532 nm	980 nm	1550 nm
α	0.53	0.51	0.70	1.20
β	-0.37	-0.34	-0.39	0.23

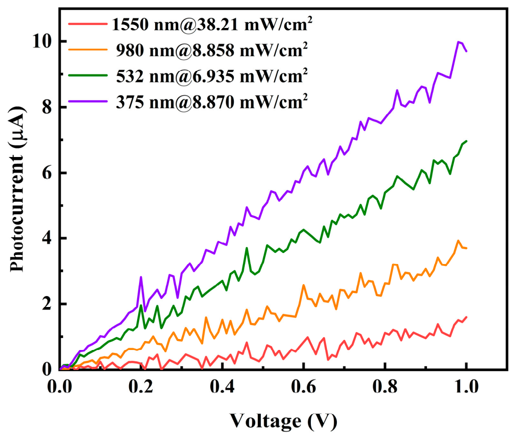

Figure S11. The photocurrent of pure graphene devices under four lasers.

Note S1 :The photoconductive gain (G) can be calculated by the following equation [1]:

$$G=\frac{\tau}{t_L}$$

The carrier transit time (t_L)is obtained from:

$$t_L = \frac{L^2}{\mu V}$$

Which is 11.49×10^{-9} s.

The photocurrent decay lifetime measurement was performed to calculate the life time of photo-excited carriers.

$$I_D = A^* \exp(-t/t_1) + I_o$$

A is a coefficient and t_1 is a constant which is considered as the lifetime of the carriers. From the curve fitting, the lifetime of the carriers is estimated to be about 0.55 s. Thus the photoconductive gain of 4.79×10^{7} can be obtained at 375 nm.

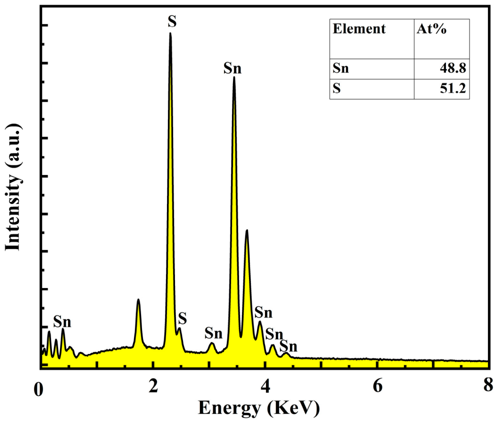

Figure S12. EDS results of the SnS nanoflakes.

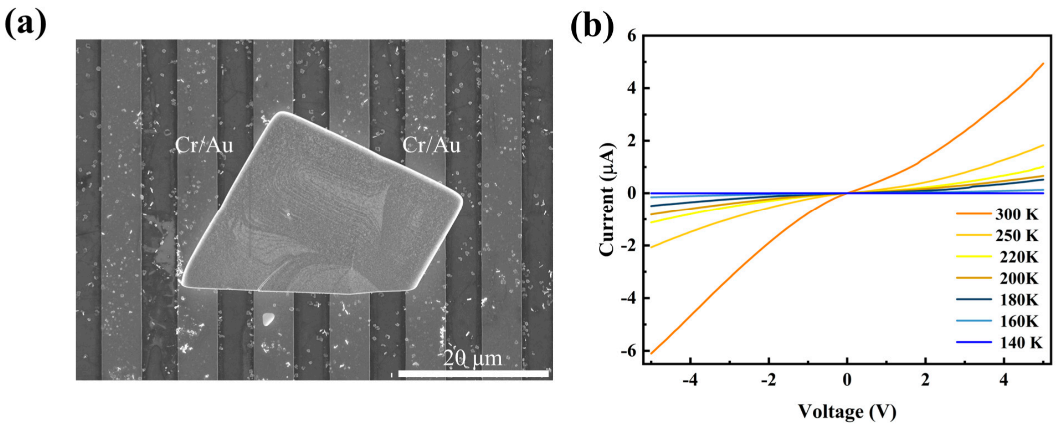

Figure S13. (**a**) The SEM image of the SnS nanoflakes FET. (**b**) The source-drain curves of the SnS nanoflakes FET were measured within the temperature range of 140 K to 300 K.

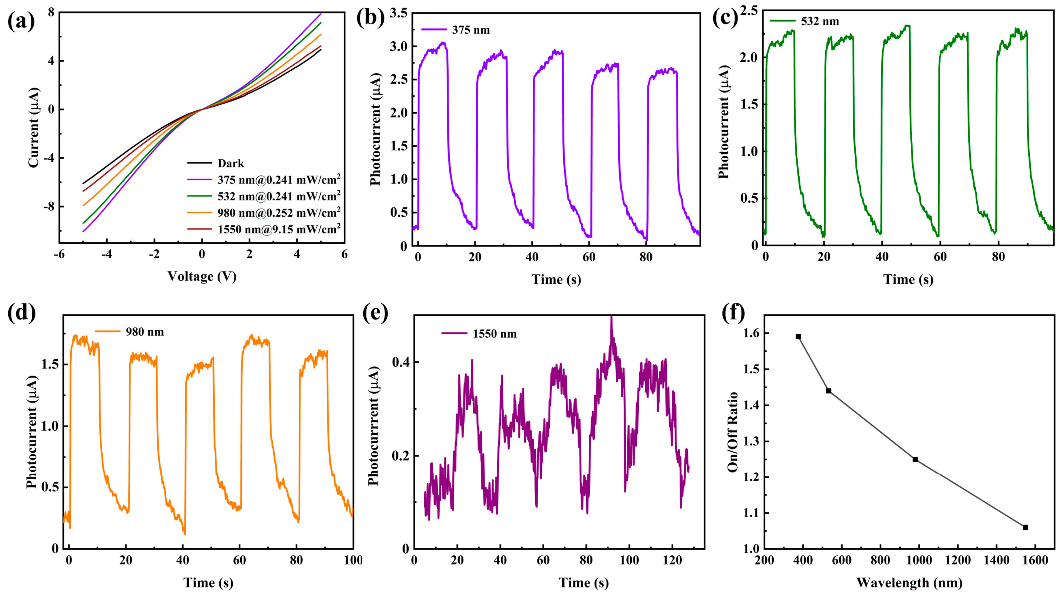

Figure 14. (**a**) IV curves of SnS FET under dark and various wavelengths of light with different light intensities. The photoresponse of the device under (**b**) 375 nm, (**c**) 532 nm, (**d**) 980 nm, (**e**) 1550 nm, and (**f**) The current on-off ratio of SnS FET under different wavelengths. (V_{DS} = 5 V).

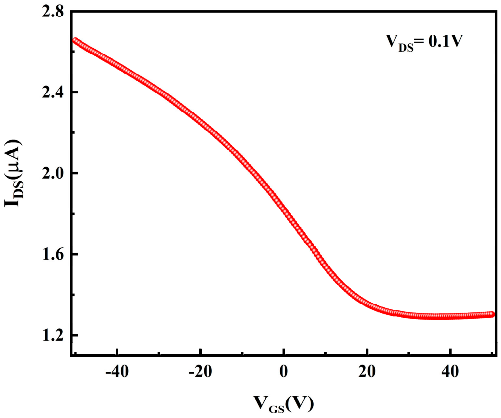

Figure S15. Transfer characteristic curves of the SnS nanoflake-based field-effect transistors at V_{DS} = 0.1 V.

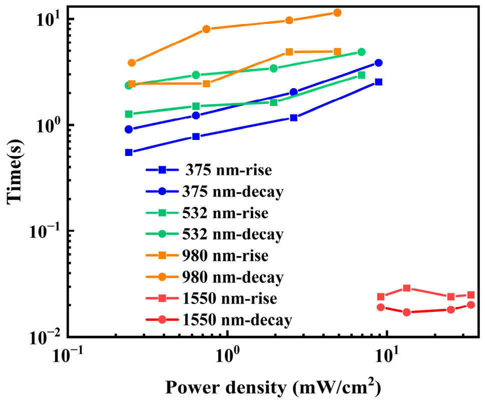

Figure S16. The power-dependent and wavelength of rise/decay response of the SnS nanoflakes/graphene devices.

Note S2: The shot noise (S_I (Shot)) can be calculated by the following equation [2]:

$$S_I\ (\text{Shot}) = 2qI_{dark}$$

The S_I (Shot) is calculated as 4.26×10^{-21} A^2/Hz.

The thermal noise (S_I (Thermal)) can be calculated by the following equation:

$$S_I\ (\text{Thermal}) = \frac{4k_BT}{R}$$

Where *R* is the resistance of the device at dark, the calculated S_I (Thermal) is 2.21×10^{-22} A^2/Hz.

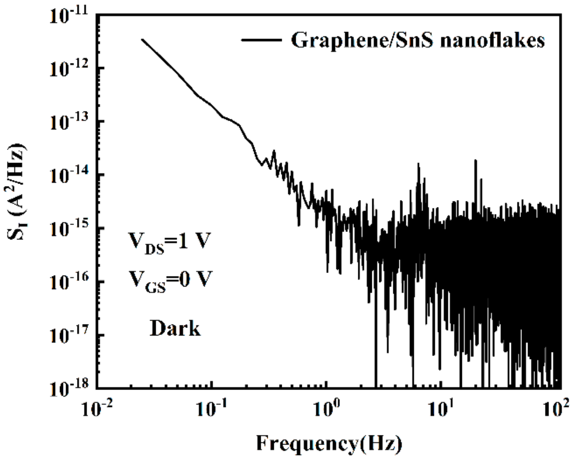

Figure S17. Noise spectral density of 1/f noise vs frequency at V_{DS} =1 V.

Figure S17 shows the 1/f noise spectral density (S_I (1/f)) of the hybrid device. The value of S_I (1/f) is 4.85×10^{-15} A^2 /Hz at a modulation frequency of 1 Hz. In summary, 1/f noise dominates the total current noise spectrum of the hybrid device.

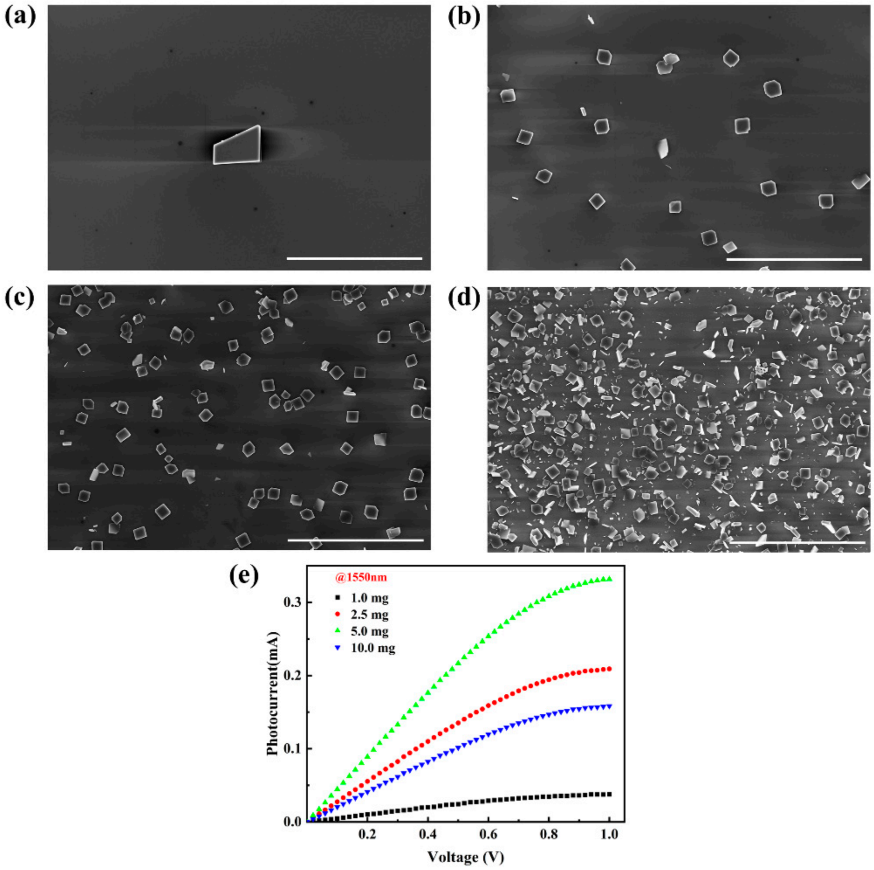

Figure S18. The SEM images of different amounts of SnS nanoflakes by varying the weight of (**a**) 1.0 mg, (**b**) 2.5 mg, (**c**) 5.0 mg and (**d**) 10 mg of the SnS powders, scale bar :10 µm. (**e**) The photocurrent of the corresponding graphene-SnS nanoflakes heterostructure phototransistor. (Wavelength: 1550 nm, 38.21 mW/cm^2).

The distribution density of SnS nanoflakes on graphene channels can be controlled by tuning the weight of SnS powder before growth. The photocurrent depends on the distribution density of SnS nanoflakes integrated with graphene, as exhibited in Figure S9e. In detail, as the powder is increased from 1 mg to 5 mg, the number of SnS nanoflakes increases, and the corresponding photocurrent also increases significantly. With regard to 10 mg, the stacking and breakage are observed in SnS nanoflakes, which is not conducive to the generation and transfer of carriers. Therefore, the photocurrent is significantly reduced for light illumination.

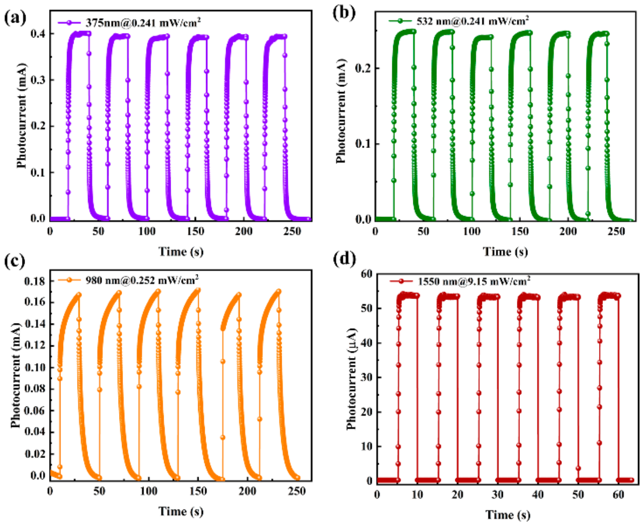

Figure S19. The photocurrent of another SnS nanoflakes/graphene devices under the laser of (**a**)375nm; (**b**)532 nm; (**c**)980 nm, and (**d**)1550 nm.

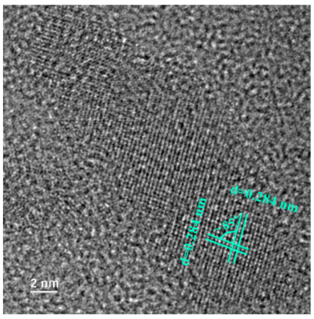

Figure S20. The HRTEM image of SnS NSs.

References:

1. Konstantatos, G.; Badioli, M.; Gaudreau, L.; Osmond, J.; Lau, S.-P.; Yan, F. Hybrid Graphene-Quantum Dot Phototransistors with Ultrahigh Gain. *Nat. Nanotechnol.* **2012**, *7*, 363–368.
2. Mukherjee, S.; Bhattacharya, D.; Patra, S.; Paul, S.; Mitra, R.K.; Mahadevan, P.; Pal, A.N.; Ray, S.K. High Responsivity Gate Tunable UV-Visible Broadband Phototransistor Based on Graphene-WS_2 Mixed Dimensional (2D-0D) Heterostructure. *ACS Appl. Mater. Interfaces* **2022**, *14*, 5775–5784.
